# Dual Leucine Zipper Kinase Is Constitutively Active in the Adult Mouse Brain and Has Both Stress-Induced and Homeostatic Functions

**DOI:** 10.3390/ijms21144849

**Published:** 2020-07-09

**Authors:** Sunil Goodwani, Celia Fernandez, Paul J. Acton, Virginie Buggia-Prevot, Morgan L. McReynolds, Jiacheng Ma, Cheng Hui Hu, Mary E. Hamby, Yongying Jiang, Kang Le, Michael J. Soth, Philip Jones, William J. Ray

**Affiliations:** 1 The Neurodegeneration Consortium, Therapeutics Discovery Division, University of Texas MD Anderson Cancer Center, Houston, TX 77054, USA; sggoodwani@mdanderson.org (S.G.); CFernandez2@mdanderson.org (C.F.); PJActon@mdanderson.org (P.J.A.); VBuggia@mdanderson.org (V.B.-P.); MLMcReynolds@mdanderson.org (M.L.M.); JMa5@mdanderson.org (J.M.); CHHu@mdanderson.org (C.H.H.); mhamby@cogrx.com (M.E.H.); 2 Institute for Applied Cancer Science, Therapeutics Discovery Division, University of Texas MD Anderson Cancer Center, Houston, TX 77054, USA; YJiang4@mdanderson.org (Y.J.); KLe3@mdanderson.org (K.L.); MJSoth@mdanderson.org (M.J.S.); PJones3@mdanderson.org (P.J.)

**Keywords:** DLK, Map3k12, cerebellum, tau, synaptic maintenance, neurodegeneration, Alzheimer’s disease

## Abstract

Dual leucine zipper kinase (DLK, Map3k12) is an axonal protein that governs the balance between degeneration and regeneration through its downstream effectors c-jun N-terminal kinase (JNK) and phosphorylated c-jun (p-c-Jun). In peripheral nerves DLK is generally inactive until induced by injury, after which it transmits signals to the nucleus via retrograde transport. Here we report that in contrast to this mode of regulation, in the uninjured adult mouse cerebellum, DLK constitutively drives nuclear p-c-Jun in cerebellar granule neurons, whereas in the forebrain, DLK is similarly expressed and active, but nuclear p-c-Jun is undetectable. When neurodegeneration results from mutant human tau in the rTg4510 mouse model, p-c-Jun then accumulates in neuronal nuclei in a DLK-dependent manner, and the extent of p-c-Jun correlates with markers of synaptic loss and gliosis. This regional difference in DLK-dependent nuclear p-c-Jun accumulation could relate to differing levels of JNK scaffolding proteins, as the cerebellum preferentially expresses JNK-interacting protein-1 (JIP-1), whereas the forebrain contains more JIP-3 and plenty of SH3 (POSH). To characterize the functional differences between constitutive- versus injury-induced DLK signaling, RNA sequencing was performed after DLK inhibition in the cerebellum and in the non-transgenic and rTg4510 forebrain. In all contexts, DLK inhibition reduced a core set of transcripts that are associated with the JNK pathway. Non-transgenic forebrain showed almost no other transcriptional changes in response to DLK inhibition, whereas the rTg4510 forebrain and the cerebellum exhibited distinct differentially expressed gene signatures. In the cerebellum, but not the rTg4510 forebrain, pathway analysis indicated that DLK regulates insulin growth factor-1 (IGF1) signaling through the transcriptional induction of IGF1 binding protein-5 (IGFBP5), which was confirmed and found to be functionally relevant by measuring signaling through the IGF1 receptor. Together these data illuminate the complex multi-functional nature of DLK signaling in the central nervous system (CNS) and demonstrate its role in homeostasis as well as tau-mediated neurodegeneration.

## 1. Introduction

Neurons, being post-mitotic cells with limited regenerative capabilities particularly in the mammalian central nervous system (CNS), are equipped with sensitive injury surveillance and response mechanisms. These mechanisms ensure that surmountable damage is not lethal, but that an irreversible lesion leads to the regulated elimination of cellular structures or even the entire neuron. One of the best characterized injury responses is Wallerian degeneration, the systematic elimination of traumatized axons, during which the distal end of the axon degrades while the proximal end is either preserved for regeneration or transmits apoptotic signals to the cell body [1,2]. Dual leucine zipper kinase (DLK), a mixed lineage kinase in the mitogen activated protein kinase (MAPK) pathway, is required for this process across species [3]. Whereas axotomy or axonal crush leads to profuse neuronal death in a number of experimental settings, in animals lacking DLK, apoptosis is delayed or prevented; in other contexts where regeneration is possible, DLK is required for the re-extension of severed axons [4,5,6,7,8,9]. 

In addition to its well-studied role in injury response, DLK is critical for nervous system development as it regulates neuronal migration [10], axon specification and neurite extension [10,11,12], axon elimination, and programmed cell death [13]. Fewer studies have examined the role of DLK in mature uninjured neurons, particularly in the mammalian CNS. In adult *Drosophila* and *C. elegans*, the DLK orthologs wallenda (Wnd) and DLK-1 regulate presynaptic function and morphology at the neuromuscular junction [14] and limit synaptic strength [15]. However, adult somatic knockout of DLK in mice revealed no CNS phenotype other than resistance to various neuronal insults and a modest increase in post-synaptic evoked potentials in the hippocampus [16]. Thus, the role of DLK, if any, in adult brain homeostasis in the absence of injury is unclear.

To perform its injury-related function, DLK activation must be permissive, as it responds to a wide array of injuries, including excitotoxicity, chemotoxicity, mechanical damage, and neurodegeneration due to pathologically misfolded and aggregated proteins or the release of apolipoprotein E [1,17,18]. At the same time, given that DLK activation can lead to axon retraction and programmed cell death, strict control over its signaling is essential. There are several possible means by which DLK signaling is restricted. One is the subcellular localization of DLK and its effectors. DLK is generally restricted to axons, growth cones, and post-synaptic densities, spatially restricting it from the cell body [8,19,20]. This compartmentalization involves the palmitoylation of DLK, which leads to physical association with transport vesicles [21]. A second mechanism is the integrity of axonal transport. When axonal transport is functioning normally, DLK remains inactive, but impaired axonal trafficking caused by cytoskeletal disruption or kinesin mutations causes the accumulation of presynaptic cargo in the axon initial segment and cell body, which triggers DLK-mediated injury response [22,23]. The abundance of DLK is also a critical factor in determining outcome. In invertebrates and mammalian peripheral nerves, DLK is constrained by rapid proteolysis resulting from ubiquitinylation by Pam/Highwire/RPM-1 (PHR) family members [12,19,24,25,26]. Injury-dependent signaling by three redundant Ste20 kinases, MAP4K4, misshapen-like kinase 1 (MINK1/Map4k6), and Traf2- and Nck-interacting kinase (TNIK/Map4k7) promote DLK stabilization [27]. Reducing DLK degradation is sufficient to trigger signaling, since blocking DLK proteolysis or overwhelming degradation by overexpression [28] are sufficient to cause pathway activation, perhaps by generating sufficient protein concentrations to drive homodimerization and intermolecular phosphorylation [29].

DLK activity is also directed to an appropriate outcome by signalsome assembly, when activated DLK interacts with its direct substrates MKK4/Map2k4 or MKK7/Map2k7, which are functionally distinct [30], and one of the three c-jun N-terminal kinase (JNK) isoforms, each of which has unique signaling properties [31]. These complexes scaffold onto one of the JNK-interacting proteins (JIPs) (JIP-1, JIP-2, JIP-3, plenty of SH3 (POSH)/Sh3rf1, and JLP/Spag9) [32,33,34,35], which are attached to motor proteins such as kinesins and provide both a platform for signaling crosstalk and a mechanism to transmit a signal to the nucleus by retrograde transport. JIPs have complex, partially overlapping binding specificities and are substrates for a number of kinases that regulate their affinity for DLK, JNK, and kinesins. The molecular composition of these retrograde signaling complexes likely impacts how signals are received and interpreted by the neuronal nucleus [36].

The ultimate output of the signaling cascade is typically induction of AP-1 transcription factors, principally c-Jun, which is phosphorylated by JNK on S63 or S73, licensing its nuclear translocation and transactivation of apoptotic or pro-regenerative genes [37]. This differential response to phosphorylated c-jun (p-c-Jun) reflects in part the availability of nuclear heterodimerization partners [38], highlighting the importance of other signaling pathways in determining the ultimate outcome of DLK activation. 

Here, we observe constitutive DLK activity leading to nuclear p-c-Jun accumulation in the adult mouse cerebellum in the absence of injury, suggesting the presence of regulatory mechanisms that permit non-apoptotic DLK signaling in mature post-mitotic neurons. To study the region-specific differences of constitutive cerebellar DLK signaling versus injury-induced DLK signaling in the forebrain, we use the rTg4510 mouse model of tauopathy. A previous study using a similar mouse model (Tau P301L) demonstrated the role of DLK in the tau-induced neuronal loss in the forebrain [18]. We compared DLK-dependent gene expression signatures in the cerebellum to those in the forebrain of non-transgenic mice, where nuclear p-c-Jun is absent, and to rTg4510 mice forebrain where DLK-dependent p-c-Jun is induced as a result of pathological tau. We found that DLK signaling elicits different downstream pathways depending on whether its function is homeostatic or tau-induced, perhaps reflecting the differential availability of scaffolding and effector proteins.

## 2. Materials and Methods

### 2.1. Study Design

C57Bl/6J male mice (3–4 months old) used in this study were obtained from the Jackson Laboratory. For all experiments DLK inhibitor (DLKi) was formulated in 0.5% methylcellulose and administered by oral gavage (10 mL/kg). Experiments utilizing DLKi were terminated 105 min following the last dose (corresponding to the time of maximal plasma and brain drug concentrations), except for the time-course experiment, and tissues were isolated as described below. For DLK antibody validation heterozygous mating of B6; 129P2-MAP3k12Gt was established to produce wild-type (*Map3k12*+/+), heterozygous (*Map3k12*+/−), and homozygous (*Map3k12*−/−) offspring. Brain tissue was extracted from E16–E20 embryos. Both rTg4510 and non-transgenic control mice were obtained from the Jackson Laboratory. All mice were initially group-housed at 22 °C with a 12-h light/dark cycle (lights on at 6 a.m.). Food and water were available ad libitum. Prior to experiments, mice were single-housed and randomly assigned to their respective treatment groups that were matched for age, sex, and littermate controls. All experimental procedures were performed according to the National Institute of Health Guidelines for the Care and Use of Laboratory Animals and were approved by the Institutional Animal Care and Use Committee of the University of Texas MD Anderson Cancer Center.

### 2.2. Tissue Harvesting

Once the mouse was deeply anesthetized with 2% isoflurane, the thoracic cavity was opened and the blood was collected using a 1 mL syringe pre-coated with heparin (Sagent Pharmaceuticals, Schaumburg, IL, USA; 5000 units/mL) and quickly transferred to an ethylenediaminetetraacetic acid, -coated Capiject (Terumo Medical Corporation, Somerset, NJ, USA) collection tube and mixed for 10 min. Isolated plasma was immediately flash frozen and stored at −80 °C until use for pharmacokinetic analysis. For the mice used in all experiments, except for ActivX KiNativ kinase profiling experiment, a cardiac perfusion was performed following blood collection using 2× PhosSTOP phosphatase inhibitors (Roche, Indianapolis, IN, USA) and 1× complete protease inhibitors (Roche) in 1× phosphate-buffered saline (PBS) pH 7.4 (Thermo Fisher Scientific, Waltham, MA, USA). The brain was removed, and the hemispheres were separated at the midline. The right hemisphere was further dissected to isolate the cerebellum, brainstem, and remaining forebrain and snap frozen. For all experiments except for RNA-Seq experiment, the left hemisphere was postfixed in 4% paraformaldehyde in PBS (Thermo Fisher Scientific) and kept at 4 °C overnight. The left hemisphere was then sequentially cryoprotected in 20% sucrose followed by 30% sucrose and maintained at 4 °C. The cryoprotected hemisphere was then sectioned sagittally at 30 µm using a cryostat (Leica Biosystems, Buffalo Grove, IL, USA). Sections were placed in cryoprotectant (30% glycerol, 30 ethylene glycol and 1× PBS) and stored at −20 °C. For RNA-Seq experiment left hemisphere was dissected to isolate the cerebellum, brainstem, and remaining forebrain and snap frozen for RNA isolation. 

### 2.3. Antibodies and DLKi

The DLKi used in this study was selected by reviewing published DLK inhibitors from patent and scientific literature and corresponds to compound 14 (Figure S1) in the article by Snehal et al. [39]. The primary antibodies used for this study were as follows: DLK, 1:250 (UC Davis/NIH NeuroMab Facility, Davis, CA, USA; clone N377/20, 75–355); DLK, 1:250 (Thermo Fisher Scientific, Waltham, MA, USA, PA532173); p-c-Jun, 1:100 (Abcam, Cambridge, MA, USA, 32385); c-Jun, 1:250 (Cell Signaling Technology, Danvers, MA, USA, 9165); glyceraldehyde 3-phosphate dehydrogenase (GAPDH), 1:5000 (MilliporeSigma, Temecula, CA, USA, MAB374); phospho-PHF-Tau Ser202/Thr205, 1:1000 (Thermo Fisher Scientific, MN1020); PSD95, 1:500 (Cell Signaling Technologies, 2507); Synaptophysin, 1:500 (Cell Signaling Technology, 4329); glial fibrillary acidic protein (GFAP), 1:500 (Cell Signaling Technology, 3670); Iba1, 1:250 (FUJIFILM Wako Pure Chemical Corporation, 01620001); p-MKK4 Ser57/Thr261, 1:100 (Cell Signaling Technology, 9156); MKK4, 1:200 (Cell Signaling Technologies, 9152); MKK7, 1:200 (Cell Signaling Technologies, 4172); JIP-1, 1:250 (Abcam, Ab24449); JIP-2, 1:250 (Abcam, Ab154090); JIP-3, 1:200 (Thermo Fisher Scientific, PA536369); POSH, 1:500 (Proteintech Group, 14649-1-AP); JNK1, 1:500 (Cell Signaling Technology, 3708); JNK2, 1:500 (Cell Signaling Technology, 9258); JNK3, 1:500 (Cell Signaling Technology, 2305); p-JNK Thr183/Tyr185, 1:500 (Cell Signaling Technology, 9251); JNK, 1:500 (Cell Signaling Technology, 9252),; IGF1 binding protein-5 (IGFBP5) 1:500 (Abcam, ab254324); p-IGF1 Y1161, 1:500 (Abcam, ab39398); IGF1, 1:500 (Abcam, ab182408); p-ERK1/2 Thr202/Tyr204, 1:500 (Cell Signaling Technology, 9101); p-AKT Ser473, 1:500 (Cell Signaling Technology, 9271); and AKT, 1:500 (Cell Signaling Technology, 4691). Goat anti-rabbit, 1:5000 (LI-COR Biosciences, Lincoln, NE, USA, 926-68021) and goat anti-mouse, 1:5000 (LI-COR Biosciences, 926-68020) were the secondary antibodies used for immunoblot detection. Primary antibodies and secondary antibodies used for immunofluorescence and immunohistochemistry staining were as follows: DLK (Thermo Fisher Scientific) and phospho c-Jun (Cell Signaling Technology). Donkey anti-rabbit and donkey anti-mouse Alexa Fluor-conjugated secondary antibodies were used for fluorescence microscopy (Thermo Fisher Scientific). 

### 2.4. Tissue Processing for Western Blot Analysis

Cerebellar, hippocampal, and anterior forebrain tissues isolated from the mice were lysed in approximately 10 volumes of radioimmunoprecipitation assay (RIPA) lysis buffer (150 mM NaCl, 1.0% IGEPAL^®^ CA-630, 0.5% sodium deoxycholate, 0.1% SDS, 50 mM Tris, pH 8.0) containing 2× Halt Protease Inhibitors (Thermo Fisher Scientific) and 2× Halt Phosphatase Inhibitors (Thermo Scientific) by mechanical homogenization on ice until a homogenous suspension was obtained. Lysates were centrifuged at 14,000× *g* at 4 °C for 30 min. The supernatants were snap frozen on dry ice and stored at −80 °C. Total protein concentration was determined using DC Protein Assay (Bio-Rad Laboratories, Hercules, CA, USA). Lysates were diluted with 4× protein sample loading buffer (LI-COR Biosciences) containing 10% 2-mercaptoethanol and heated to 95 °C for 10 min. The samples were then subjected to gel electrophoresis on NuPAGE 4–12% Bis-Tris Gels (Thermo Fisher Scientific) using 1× MES running buffer (Thermo Fisher Scientific) after which they were transferred to nitrocellulose membranes (Thermo Fisher Scientific) and incubated with Odyssey Blocking Buffer (LI-COR Biosciences) for 1 h at room temperature. The membranes were then incubated with primary antibodies overnight at 4 °C with continual shaking, washed three times with 1× tris-buffered saline –0.1% Tween (TBS-T) at room temperature with continuous shaking, and then incubated in secondary antibodies for 1 h at room temperature with continuous shaking before washing with TBST three times. The membranes were then imaged using the Odyssey CLx Imaging System (LI-COR Biosciences, Lincoln, NE, USA) and quantified using Image Studio 4.0 Software (LI-COR Biosciences). The data were analyzed using Microsoft Excel and GraphPad Prism (GraphPad Software). Cytosolic and nuclear fractions from cerebellum were extracted using Thermo Scientific™ NE-PER™ Nuclear and Cytoplasmic Extraction Reagents (as per the manufacturer’s protocol).

### 2.5. Immunohistochemistry and Immunofluorescence Analysis

Free floating sagittal brain sections (30 µm) were washed in TBS and subjected to antigen retrieval for 20 min at 90 °C in a sodium citrate buffer (Sigma-Aldrich, St. Louis, MO, USA). For immunofluorescence staining endogenous fluorescence was quenched by incubation in 10 mM glycine in TBS with 0.25% Triton X-100 (TBS-T) followed by blocking in 5% horse serum in TBS-T. Sections were incubated overnight at 4 °C in primary antibodies diluted in 1% bovine serum albumin (BSA) in TBS-T, followed by secondary antibody incubation for 2 h at room temperature. Sections were stained for nuclei using Hoechst 33,342 (Thermo Fisher Scientific), mounted on Superfrost Plus slides (Thermo Fisher Scientific) in VectaShield mounting media (Vector Laboratories, Burlingame, CA, USA) and allowed to cure overnight before imaging. DAB (3,3’-diaminobenzidine) immunohistochemistry was performed using Vector ABC (Vector Laboratories) according to the manufacturer’s instructions. After the final reaction was terminated, sections were mounted on Superfrost Plus slides in Cytoseal XYL mounting media (Sigma) and allowed to cure overnight before imaging. Images were acquired on a Nikon Eclipse Ti confocal microscope using 20× and 60× objectives and NIS Elements Imaging Software (Nikon Instruments, Melville, NY, USA). For comparison between conditions, the same acquisition settings were used for each channel across samples. All images were processed using ImageJ software (NIH), using the signal from control IgG staining to set the background. To assess the level of p-c-Jun immunofluorescence in Hoechst-positive nuclei in the brain, a mask was created in the Hoechst image, applied to the corresponding p-c-Jun image, and fluorescence outside the region defined by the mask was cleared. The remaining fluorescence was quantified by measuring the raw integrated density in the field, which is the sum of the pixel values in the image. The raw integrated density signal was then expressed as a percentage of the signal in the cerebellum of vehicle-treated control.

### 2.6. RNA-Seq Analysis

RNA was isolated from the cerebellum and forebrain using Qiagen RNeasy Mini kit and Qiagen TissueRuptor (Qiagen, Germantown, MD, USA) per the manufacturer’s recommendations. mRNA-Seq was performed using Illumina HiSeq 4000 with 76 paired-end reads. Sequence read quality was assessed using FastQC v0.11.5 (https://www.bioinformatics.babraham.ac.uk/projects/fastqc/) before and after adapter trimming. Adapter trimming was conducted using Cutadapt v1.15 (https://cutadapt.readthedocs.io/en/v1.15/). The trimmed paired-end read sequences were then aligned to the mouse reference genome GRCm38 (mm10) using the STAR aligner v2.5.1b [40,41]. Genes were quantified using the STAR aligner option quantMode which utilizes the HTSeq algorithm [42]. Low counts were discarded, and data were normalized (standard method), followed by differential gene expression analysis (ANOVA) using Partek Flow version 7.0 (Partek Incorporated, St. Louis, Missouri, USA). Heatmap was generated using Heatmapper [43]. Distant regulatory elements of co-regulated genes (DiRE) was used for promoter analysis [44].

### 2.7. Pharmacokinetic Assessment of DLKi in Plasma and Brain Tissue

Concentration of DLKi in both plasma and brain tissue samples was quantified by LC-MS/MS after sample processing. For plasma sample analysis, 25 μL of each sample was precipitated with 200 μL of acetonitrile containing 5 ng/mL of a reference compound that served as the internal standard (IS). This suspension was vortexed for 30 min and centrifuged at 4000 rpm for 15 min, after which 100 μL of the extract was diluted with 200 μL of water and mixed prior to LC-MS/MS analysis. For brainstem tissue sample analysis, the tissue samples were homogenized in 80% methanol/water (1 mL per 100 mg tissue) at 4 °C using OMNI Bead Ruptor 24 coupled with Omini BR-Cryo cooling unit (Omni International, Kennesaw, GA, USA). After centrifugation at 15,000 rpm for 15 min, the supernatant (60 μL) was mixed with 50% acetonitrile/water (140 µL) containing 5 ng/mL internal standard prior to LC-MS/MS analysis. LC-MS/MS analysis was conducted on a Waters Acquity (ultra performance liquid chromatography (UPLC) system coupled with a Waters TQ-S triple quadrupole mass spectrometer system operated at positive mode (Waters, Manchester, UK). The DLKi and IS were separated using a Supelco Ascentis fused-core C18 column (2.7 μm, 2.1 × 20 mm) (Sigma-Aldrich, St. Louis, MO, USA) and detected by multiple reaction monitoring transitions. The mobile phase A was 0.1% acetic acid-water and B was 0.1% acetic acid-acetonitrile. The gradient was 15% B (0–0.3 min), 15–95% B (0.3–1.3 min), 95–15% B (1.3 to 1.31 min), 15% B (1.31 to 1.7 min), and the flow rate was 0.5 mL/min. The column temperature was 40 °C. The injection volume was 2 µL. Under these conditions, the retention time was 0.8 min for DLKi and 1.08 min for IS. The method was validated with an analytical range of 1–1000 ng/mL DLKi in both untreated mouse plasma and brain tissue homogenate.

### 2.8. Statistical Analysis

The data is expressed as mean ± SEM plotted using GraphPad Prism software (GraphPad Software). Statistical analysis of the differences between groups was performed using Student’s unpaired *t*-test when comparing two groups, one-way repeated measures ANOVA followed by Tukey’s multiple comparisons test when comparing more than two groups, or one-way repeated measures ANOVA, followed by Dunnett’s *t*-test when comparing multiple groups to one control group. Pharmacokinetic (PK)/pharmacodynamic analysis was performed using GraphPad Prism software. The percent change in cerebellar p-c-Jun/c-Jun levels relative to vehicle control obtained from the Western blot analysis was utilized as the pharmacodynamic response and plotted against the PK parameter. Non-linear regression analysis followed by variable slope (four parameters) was used to calculate IC_50_ of DLKi in Figures S3 and S4. Pearson correlation analysis was performed to determine the correlation of p-c-Jun levels with GFAP, IBA1, SYNAP, PSD95, and p-TAU AT8, as shown in; *p* < 0.05 was considered statistically significant.

## 3. Results

### 3.1. Brain Region-Dependent Constitutive or Induced Phosphorylation of c-Jun

**Figure 1:**
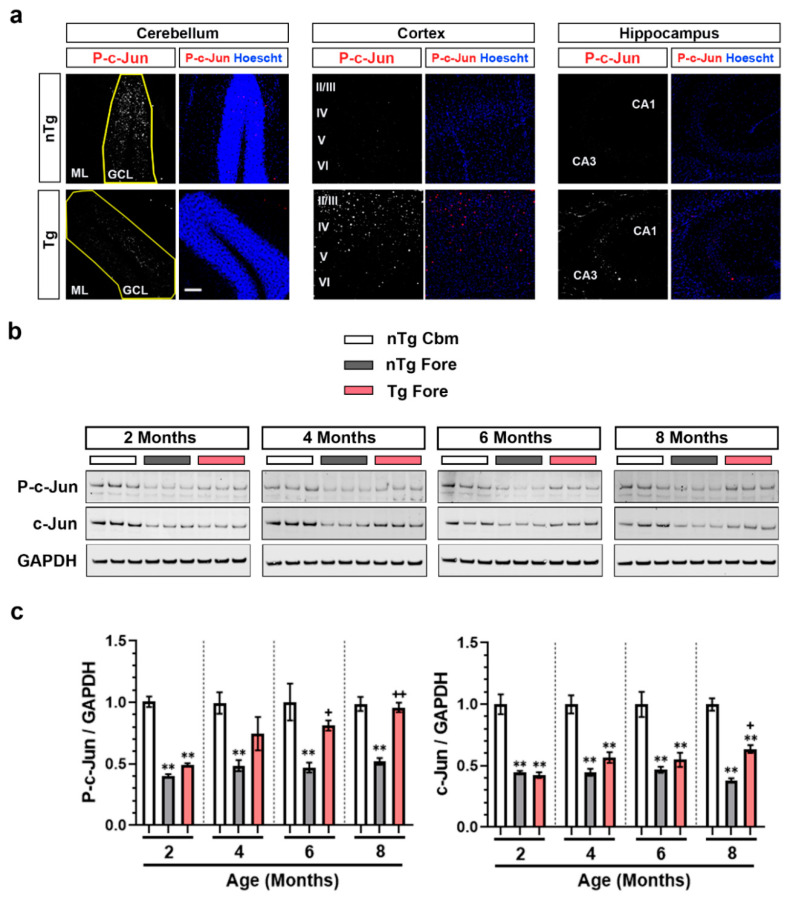
Phosphorylated c-jun (P-c-jun) expression in cerebellum versus forebrain of nTg and Tg mice. (**a**) Representative images of p-c-Jun and Hoechst co-staining in the cerebellum, cortex, and hippocampus of 7–8 months old female nTg and Tg mice. Scale bar: 100 mm. (**b**) Representative immunoblots of p-c-Jun and c-Jun of protein lysates obtained from nTg cerebellum (nTg Cbm), nTg forebrain (nTg Fore), and Tg forebrain (Tg Fore) of 2, 4, 6, and 8 months old female mice. (**c**) Quantification of p-c-Jun/GAPDH and c-Jun/GAPDH of the immunoblots from (b), plotted as mean fold change relative to nTg cerebellum group of respective age cohort. One-way repeated measure ANOVA followed by Tukey’s multiple comparisons test. ** *p* < 0.01 relative to nTg cerebellum, * *p* < 0.05, + *p* < 0.05, ++ *p* < 0.01 relative to nTg forebrain, *n* = 6.

In the adult nervous system, DLK is generally regarded as injury-induced, and its activation contributes to synapse elimination and neuronal loss [18]. To characterize DLK-dependent p-c-Jun in a mouse model of tauopathy, we examined female rTg4510 mice. These mice express high levels of P301L mutant human tau specifically in the forebrain, causing widespread neuronal loss in an age-dependent manner by 6 months of age, but sparing the cerebellum [45]. A similar mouse model (Tau P301L) was used to demonstrate that DLK is required for nuclear p-c-Jun accumulation and contributes to neuronal loss in the subiculum [18]. Immunofluorescence staining of p-c-Jun in sagittal brain sections from ~8 months old rTg4510 (Tg) mice and non-transgenic littermates (nTg) allowed us to compare the cortex and hippocampus in the forebrain, as well as cerebellum (non-injured brain region) in the same section of tissue. Consistent with previous reports in Tau P301L mice, we observed an increase in p-c-Jun positive cells in the hippocampus and cortex of Tg mice relative to nTg mice (Figure 1a). Interestingly, we also observed high basal p-c-Jun staining in both Tg and nTg cerebellum. 

We then performed Western blotting on the forebrain and cerebellar lysates from 2, 4, 6, and 8 months old nTg and Tg mice, and compared p-c-Jun levels. In line with our immunofluorescence data, Western blot analysis confirmed that Tg mice, unlike nTg, showed an age-dependent increase in p-c-Jun in the forebrain, reaching significance at 6 months of age (*p* < 0.01) (Figure 1b,c). In nTg mice, the cerebellum had significantly higher levels of p-c-Jun compared to forebrain at all ages measured. p-c-Jun in Tg forebrain, compared to nTg cerebellum was significantly lower at 2 months of age, however this difference diminished with induction of p-c-Jun in Tg forebrain beginning at 4 months of age. Thus p-c-Jun is constitutively expressed in cerebellum but induced in the forebrain during the disease process. 

**Figure 2:**
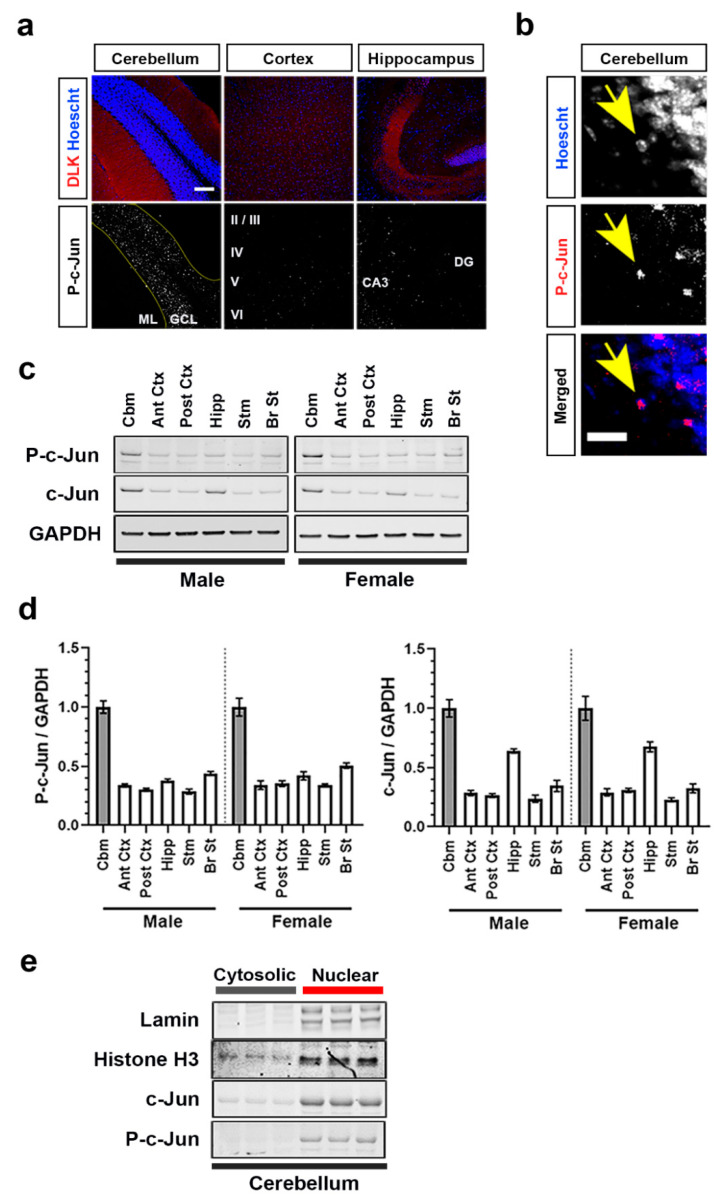
Dual leucine zipper kinase (DLK), p-c-Jun, and c-Jun expression in adult uninjured mouse brain. (**a**) Representative double staining for DLK (red) and Hoechst (blue) shows DLK’s distribution in the molecular layer of the cerebellum, diffusely throughout the cortex and in the mossy fiber axon terminals in the hippocampus of 3–4 months old male wild-type (WT) mouse brain. Representative staining for p-c-Jun shows its distribution in the granule cell layer of the cerebellum, in layer V of the cortex and to a lesser degree in the CA3 region of the hippocampus. GCL, granule cell layer; ML, molecular layer; CA3, Cornu Ammonis subfield 3 of the hippocampus; DG, dentate gyrus. Scale bar: 100 µm. (**b**) p-c-Jun (red) fluorescence overlaps with Hoechst (blue)-positive nuclei, indicating a nuclear localization. Scale bar: 20 µm. (**c**) Immunoblots of p-c-Jun and c-Jun in lysates obtained from cerebellum (Cbm), anterior cortex (Ant Ctx), posterior cortex (Post Ctx), hippocampus (Hipp), striatum (Stm), and brainstem (Br St) of 3–4 months old male and female WT mice. (**d**) Quantification of p-c-Jun/GAPDH and c-Jun/GAPDH immunoblots in (c) (represented as fold change relative to cerebellar levels). (**e**) Immunoblots of Lamin, Histone H3, p-c-Jun, and c-Jun in cytosolic and nuclear fractions of 3–4 months old male WT mice cerebellum.

To study this constitutive cerebellar p-c-Jun in fully wild-type (WT) mice, we next examined the uninjured male C57Bl/6J mouse brain. We first assessed DLK expression in the cerebellum, forebrain, and hippocampus of these mice using immunofluorescence staining (Figure 2a). The specificity of the DLK antibody was confirmed using DLK −/− embryonic mouse brains (Figure S2a). In each region of the WT mouse brain, DLK immunoreactivity broadly labeled neurons and their processes, with particular intensity in the molecular layer of the cerebellum and in the mossy fibers extending to the CA3 region of the hippocampus (Figure 2a). Morphological examination revealed that DLK is predominantly expressed in axons, consistent with previous reports [46]. Next, we examined p-c-Jun; these WT mice were handled for 4 days prior to sacrifice to prevent stress-induced phosphorylation of c-Jun. Intense immunoreactivity for p-c-Jun was noted in the nuclei in the granule cell layer of the cerebellum within cells that have the morphological appearance of cerebellar granule neurons (Figure 2a,b). In contrast, p-c-Jun staining was sparse in other brain regions with only rare p-c-Jun-positive nuclei. Next we compared DLK, p-c-Jun, and c-Jun levels in the cerebellum, anterior forebrain, posterior forebrain, hippocampus, striatum, and brain stem of WT mice. The antibody specifically detected a band at 120 kD that was absent in the DLK −/− mouse brain lysate (Figure S2b). Using this validated DLK antibody, we found that DLK levels across all the brains regions from WT mice were comparable, with the cerebellum expressing ~20% more DLK compared to the rest of the brain regions analyzed (Figure S3a,b). The biochemical assessment of p-c-Jun aligned with the immunofluorescence data: levels were approximately 6-fold higher in the cerebellum than in rest of the brain regions (Figure 2c,d). As would be predicted since p-c-Jun drives the transcription of *Jun*, c-Jun levels were also higher in the cerebellum [47]. This increase in p-c-Jun in the cerebellum was not sex-specific as it was observed in both male and female mice. To confirm the subcellular location of cerebellar p-c-Jun, we isolated nuclear and cytosolic fractions. The purity of the subcellular fractions were confirmed by immunoblotting for Lamin B and Histone H3, which are nuclear proteins (Figure 2e). In the cerebellum of WT mice, while most of the c-jun was nuclear, some c-jun were cytosolic. On the contrary, p-c-Jun was almost exclusively observed in nuclear extracts, confirming the immunostaining results. 

### 3.2. Constitutive Cerebellar p-c-Jun and tau-Induced Forebrain p-c-Jun Are Both DLK Dependent

**Figure 3:**
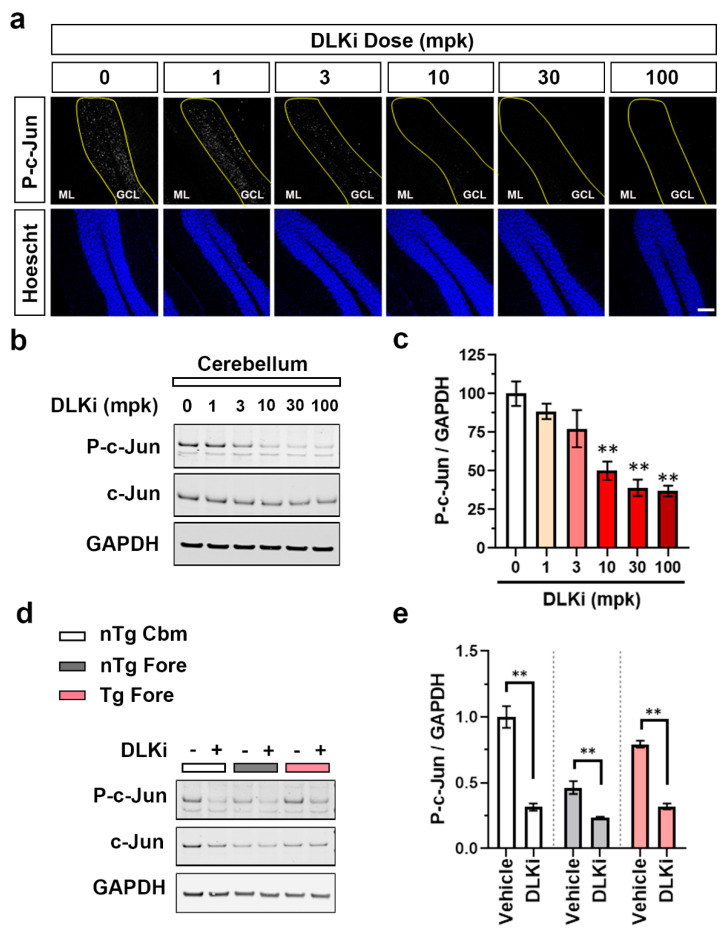
Pharmacological inhibition of DLK inhibits constitutive as well as induced p-c-Jun in adult mouse brain. (**a**) Representative staining for p-c-Jun and Hoechst (blue) in the cerebellum of 3–4 months old WT mice dosed with a single oral dose of vehicle or DLK inhibitor (DLKi) at 1, 3, 10, 30, or 100 mg/kg. (**b**) Representative immunoblots of p-c-Jun and c-Jun in cerebellar homogenates of 3–4 months old male WT mice dosed with a single oral dose of vehicle or DLKi at 1, 3, 10, 30, or 100 mg/kg. (**c**) Quantification of immunoblots of p-c-Jun/GAPDH in cerebellar homogenates of 3–4 months old male WT mice dosed with a single oral dose of vehicle (*n* = 7) or DLKi at 1 (*n* = 4), 3 (*n* = 4), 10 (*n* = 4), 30 (*n* = 4), and 100 (*n* = 4) mg/kg, plotted as percent mean fold change relative to vehicle-treated group. ** *p* < 0.01, one-way repeated measures ANOVA, followed by Dunnett’s *t*-test. (**d**) Representative immunoblots of p-c-Jun and c-Jun in homogenates from nTg cerebellum (nTg Cbm), nTg forebrain (nTg Fore), and Tg forebrain (Tg Fore) of 7–8 months old female mice dosed with 50 mg/kg of DLKi or vehicle QD for 7 days. (**e**) Quantification of the immunoblots of p-c-Jun/GAPDH from homogenates obtained from nTg cerebellum, nTg forebrain, and Tg forebrain of mice treated with vehicle or DLKi, plotted as mean fold change relative to vehicle group in respective brain region. *n* = 6, ** *p* < 0.01, unpaired *t*-test.

Given this observation, next we asked whether the DLK in the axons of cerebellar granule cells drives the phosphorylation and nuclear accumulation of c-Jun in the absence of injury. To block DLK activity fully throughout the brain, we used a highly selective DLK inhibitor (DLKi) with good oral bioavailability and CNS penetration [39]. Acutely blocking DLK rather than relying on conditional knockouts has two advantages: complete DLK inhibition is possible, and the short duration of action minimizes the potential for compensation. This molecule, designated compound 14 (Figure S1), is a potent (IC_50_ = 3 nM) ATP site-directed small molecule that has good oral bioavailability and mouse pharmacokinetics, and importantly, has very high in vitro binding selectivity for DLK with no sub-micromolar inhibition of any other kinase detected in a broad kinome panel other than DLK’s close homolog LZK, for which it was 40-fold selective. We confirmed the potency, selectivity, and pharmacokinetic profile of this DLKi prior to utilizing it for in vivo studies. We administered DLKi orally to male WT mice and assessed cerebellar p-c-Jun levels by Western blot and immunofluorescence. At 105 min post single dose of DLKi, nuclear p-c-Jun immunofluorescence in the granule cell layer was nearly completely abolished in a dose-dependent manner (Figure 3a). Western blots of cerebellar lysates indicated that DLK protein levels were unchanged, but p-c-Jun levels declined 61–63% in 30 mg/kg and 100 mg/kg DLKi-treated mice compared to vehicle-treated mice (Figure 3b,c) (*p* < 0.01). This rapid reduction in p-c-Jun occurred as a function of total DLKi concentration in plasma and brain (Figure S4a) and the IC_50_ for the DLKi was calculated to be 1.57 μM in plasma and 2.59 μM in the brain. Using the reported protein binding of 86.8% in plasma and 93.2% in the brain we calculated free concentration of this DLKi in both compartments and plotted it against the p-c-Jun (Figure S4b), and calculated the IC_50_ to be 0.207 μM in plasma and 0.176 μM in the brain [39]. These numbers align closely with the potency for DLKi in cellular assays [39]. 

To confirm the selective action of the DLKi against the kinome in vivo, we used the KiNativ in-situ kinase profiling assay, a mass-spectrometry-based target engagement method that measures all the kinase(s) a drug has bound in tissue [48]. KiNativ in situ kinase profiling of WT brain tissue harvested 105 min after dosing with DLKi at doses that significantly reduce p-c-Jun levels demonstrated that of the 242 kinases detected, only DLK was fully bound by the drug (Figure S5a). At 5 mg/kg, 15 mg/kg, and 50 mg/kg doses, DLK was approximately 40%, 60%, and >80% bound, respectively, to DLKi in the brain (Figure S5b). Furthermore, the plotting percent DLK binding against total and free brain concentration of DLKi revealed IC_50_ of 3.97 μM and 0.27 μM, respectively (Figure S5c); thus, DLK target engagement IC_50_ values are very similar to the p-c-Jun IC_50_ values. Thus, we conclude that DLK drives the majority of the constitutively expressed p-c-Jun in the cerebellum. 

Next we evaluated if the pathologically induced p-c-Jun in the Tg forebrain was inhibited by DLKi. To determine the optimal dose for this study we administered a single oral dose of DLKi (50 mg/kg) or vehicle to adult male WT mice and isolated the brain tissue at 2, 4, 8, 12, and 24 h later (Figure S6a). Pharmacokinetic analysis revealed that the concentration of DLKi in the brain remained above the IC_50_ for 24 h after a single 50 mg/kg (PO) oral dose (Figure S6b). Therefore, 50 mg/kg DLKi QD (once a day) provided sufficient exposure (≥IC_50_) to inhibit the pathway. 

We then dosed 7–8 months old nTg and Tg mice for 7 days with DLKi or vehicle QD and examined brain tissue by Western blot (Figure 3d). As with the previous experiments, p-c-Jun in the nTg cerebellum decreased 68.4% with DLKi treatment as compared to the vehicle (*p* < 0.01) (Figure 3e). In the nTg forebrain, the low baseline level of p-c-Jun was even further reduced by DLKi by 22.8% (*p* < 0.01), suggesting that even in the absence of pathology, DLK has some low level of constitutive activity in that brain region. In the forebrain of the Tg mice, DLKi reduced p-c-Jun by 47.4% (*p* < 0.01), fully eliminating the p-c-Jun induced by pathological tau. Therefore, like constitutively-expressed cerebellar p-c-Jun, tau-induced p-c-Jun in the forebrain is also nearly entirely DLK-dependent.

### 3.3. DLK Is Constitutively Active in Adult Mouse Brain

**Figure 4:**
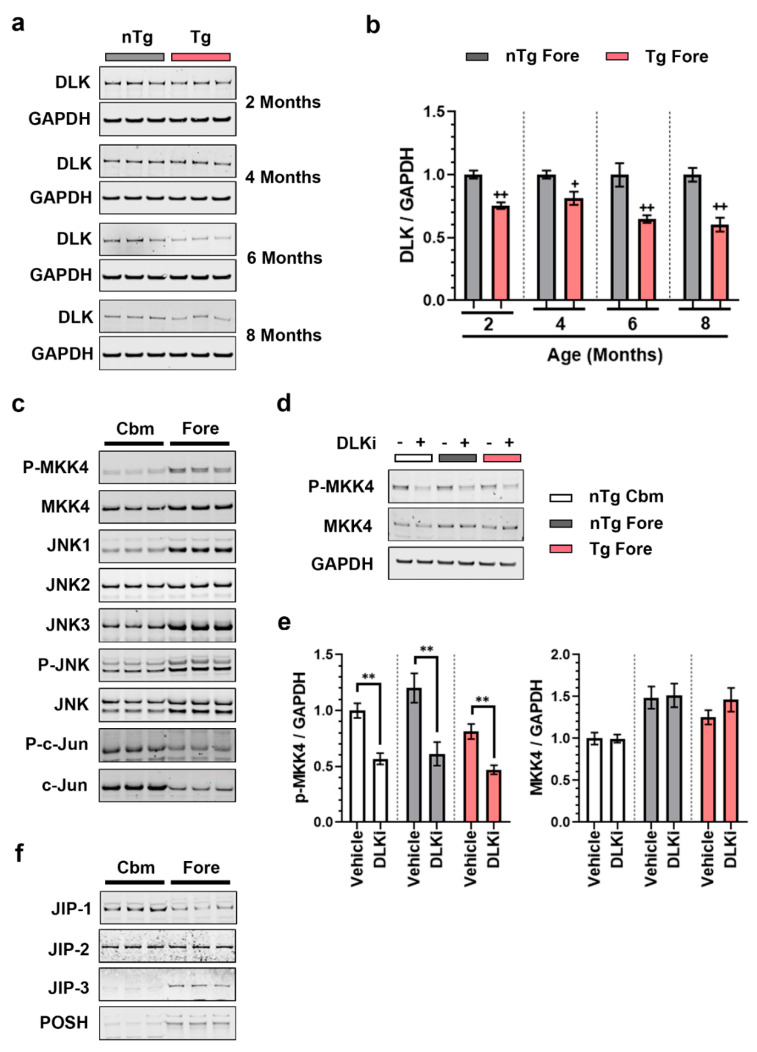
DLK is constitutively active in all the brain regions of adult mouse brain but has brain region specific scaffolding proteins. (**a**) Representative immunoblots of DLK from the homogenates obtained from nTg and Tg forebrain of 2, 4, 6, and 8 months old female mice. (**b**) Quantification of the immunoblots of DLK/GAPDH from the nTg and Tg forebrain homogenates from 2, 4, 6, and 8 months old female mice, plotted as mean fold change relative to nTg group of respective age cohort. Unpaired *t*-test. + *p* < 0.05, ++ *p* < 0.01, *n* = 6. (**c**) Immunoblots showing differential expression of DLK signaling components: p-MKK4, MKK4, JNK1, JNK2, JNK3, p-JNK, JNK, p-c-Jun, and c-Jun from cerebellum (Cbm) and forebrain (Fore) from 3–4 months old male WT mice. (**d**) Representative immunoblots of p-MKK4 and MKK4 in homogenates from nTg cerebellum (nTg Cbm), nTg forebrain (nTg Fore), and Tg forebrain (Tg Fore) of 7–8 months old female mice treated with 50 mg/kg of DLKi or vehicle QD for 7 days. (**e**) Quantification of the immunoblots of p-MKK4/GAPDH and MKK4/GAPDH from homogenates obtained from nTg cerebellum, nTg forebrain, and Tg forebrain of mice treated with vehicle or DLKi, plotted as mean fold change relative to vehicle group in the respective brain region. Unpaired *t*-test. ** *p* < 0.01, *n* = 6. (**f**) Immunoblots showing differential expression of DLK scaffolding proteins: JIP1, JIP2, JIP3, and POSH from cerebellum (Cbm) and forebrain (Fore) from 3–4 months old male WT mice.

To understand the mechanism by which nuclear p-c-Jun is increased in Tg forebrain, we examined the DLK signaling pathway by Western blot analysis of nTg and Tg forebrain lysates from 2, 4, 6, and 8 months old mice. In contrast to observations in nerve injury models, where stabilization of DLK is linked to its signaling activity, increased p-c-Jun in the Tg forebrain did not coincide with increased DLK protein levels (Figure 4a,b); on the contrary, DLK protein levels were lower in the Tg forebrain, perhaps due to the axonal loss in this model. Since DLK stabilization did not explain the increased p-c-jun, we next examined MKK4 and MKK7, which are the direct substrates of DLK, the three JNK isoforms, c-Jun, and the relevant phosphorylated forms of these proteins when possible. In adult WT mouse cerebellum and forebrain (Figure 4c), MKK4, p-MKK4, JNK1, JNK3, and total p-JNK protein levels were all higher in the forebrain than in the cerebellum in WT mice, while before, p-c-Jun levels were higher in the cerebellum. MKK7 total protein did not differ by brain region, while pMKK7 was undetectable with available antibodies. Thus, the low nuclear p-c-Jun in the uninjured forebrain is not due to the absence of DLK signaling pathway components; conversely many of these proteins are higher in the forebrain. 

Since p-MKK4 was observed in the cerebellum and particularly in the forebrain, we asked if its phosphorylation was DLK-dependent. Western blot analysis of mice treated with 50 mg/kg of DLKi QD for seven days revealed that DLKi decreased p-MKK4 levels in nTg cerebellum, nTg forebrain, and Tg Forebrain by 50–60% compared to vehicle-treated mice (*p* < 0.01) (Figure 4d,e). MKK7 levels were unchanged. Therefore, DLK activity is required for the constitutive expression of p-MKK4 in all three contexts. Together these data further suggest that the differences in p-c-Jun are not due to the expression of DLK or its signaling partners, nor the activity of DLK itself.

Since DLK/JNK signaling components are assembled into multi-protein signalosomes, we hypothesized that the availability of scaffolding proteins in the cerebellum versus forebrain could potentially drive the differences in DLK-dependent c-Jun phosphorylation. We examined our RNA-Seq data (see below) for regional disparities in expression of known JNK scaffolding genes. Of the genes examined, only one, *Sh3rf1* (POSH), is highly regionally enriched, with >10-fold greater expression in the forebrain than in the cerebellum (Figure S7). Thus, we performed Western blot analysis for JIP-1, -2, -3, and POSH. In the WT mouse brain, JIP-1 is predominantly expressed in the cerebellum as previously reported [49], whereas JIP-3 and POSH are expressed relatively higher in the forebrain, and are nearly undetectable in the cerebellum (Figure 4f). Thus, the cerebellum and forebrain have different scaffolding proteins available for DLK signalsome formation. 

### 3.4. Forebrain p-c-Jun Correlates with Markers of Neuronal Injury in rTg4510 Mouse Model

**Figure 5:**
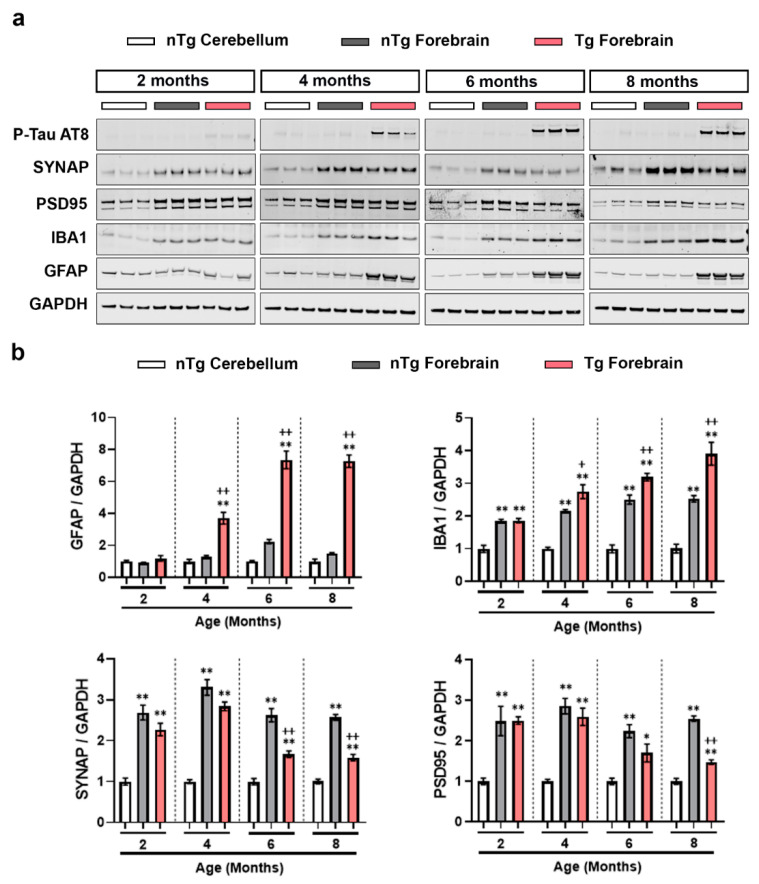
Expression of neuronal injury markers and synaptic markers in rTg4510 mouse brain. (**a**) Representative immunoblots of p-TAU AT8, SYNAP, PSD95, GFAP, and IBA1 from the homogenates obtained from nTg cerebellum as well as nTg and Tg forebrain of 2, 4, 6, and 8 months old female mice. (**b**) Quantification of the immunoblots of GFAP/GAPDH, IBA1/GAPDH, SYNAP/GAPDH and PSD95/GAPDH, from the homogenates obtained nTg cerebellum, nTg forebrain and Tg forebrain of 2, 4, 6, and 8 months old female mice, plotted as mean fold change relative to nTg cerebellum group of respective age cohort. One-way repeated measure ANOVA followed by Tukey’s multiple comparisons test. * *p* < 0.05, ** *p* < 0.01 relative to nTg cerebellum; + *p* < 0.05, ++ *p* < 0.01 relative to nTg forebrain, *n* = 6.

We next wanted to understand how induction of p-c-Jun in the forebrain of the Tg mice related to the onset of pathology, therefore we compared it to markers of neurodegeneration over time. Immunoblotting with AT8 antibody confirmed that Tg forebrain exhibited age-dependent accumulation of hyperphosphorylated tau (Figure 5a,b). As expected, cerebellar and forebrain lysates from nTg mice had negligible amounts of hyperphosphorylated tau. Next we examined markers of neuronal injury and synaptic proteins and, as expected, observed increases in the markers of gliosis, GFAP, and IBA1, denoting astrocytic and microglial activation, and reduction in the synaptic proteins synaptophysin (SYNAP, a presynaptic protein) and PSD-95 (a post-synaptic protein) compared to nTg forebrain. 

**Figure 6:**
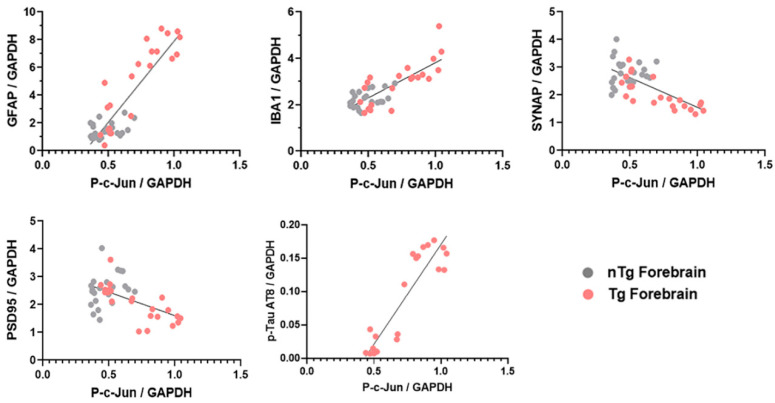
Correlation of p-c-Jun with neuronal injury markers and synaptic markers in rTg4510 mouse brain. Correlation plots between the amount of GFAP/GAPDH (R^2^ = 0.78), IBA1/GAPDH (R^2^ = 0.63), SYNAP/GAPDH (R^2^ = 0.46), PSD-95/GAPDH (R^2^ = 0.28), and p-TAU AT8/GAPDH (R^2^ = 0.83) with p-c-Jun/GAPDH in forebrain of nTg and Tg mice. Grey circles, nTg; pink circles, Tg; *p* < 0.0001, Pearson correlation.

Next we compared the relative protein expression of these disease state markers with p-c-Jun levels from nTg and Tg mice forebrain (Figure 6). Pearson correlation analysis of GFAP and IBA1 revealed a positive correlation with p-c-Jun in the forebrain (R^2^ = 0.884, *p* < 0.0001 for GFAP and R^2^ = 0.796, *p* < 0.0001 for IBA1). Furthermore, Pearson correlation analysis of the synaptic proteins, synaptophysin, and PSD95, revealed a negative correlation with p-c-Jun in the forebrain (R^2^ = −0.682, *p* < 0.0001 for synaptophysin and R^2^ = −0.531, *p* = 0.0001 for PSD-95). Furthermore, Pearson correlation analysis of hyperphosphorylated tau revealed a significant positive correlation with p-c-Jun in the forebrain of the Tg mice (R^2^ = 0.913, *p* < 0.0001). Taken together, these data suggest that in the forebrain, unlike the cerebellum, p-c-Jun coincides with pathological progression and disease severity in the Tg mice. 

### 3.5. Downstream Consequences of DLK Activity Are Regionally Distinct

**Figure 7:**
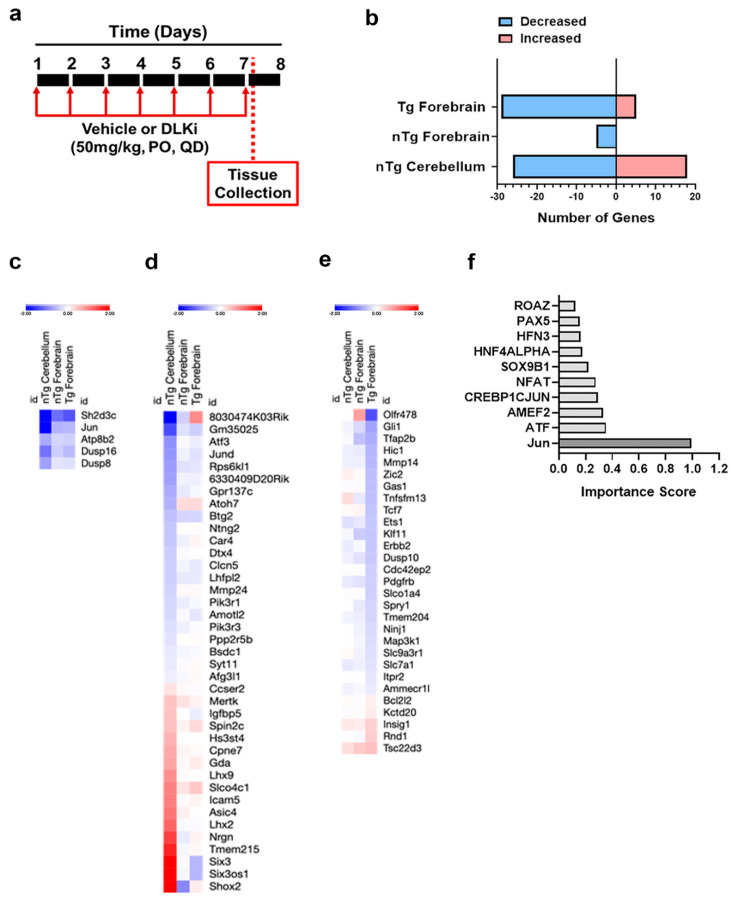
RNA-Seq analysis reveals a DLK-dependent gene signature in both the cerebellum and forebrain in nTg and Tg mice. (**a**) Schematic representation of study timeline. nTg (*n* = 6) and Tg (*n* = 6) mice were treated with 50 mg/kg of DLKi or vehicle QD for 7 days. The study was terminated approximately 2 h after the last dose. (**b**) Number of differentially expressed genes (red = increased, blue = decreased; *p*_adj_ < 0.05) due to DLK inhibition for each brain region × genotype is plotted. (**c**) Heatmap depicting the more robustly altered genes (*p*_adj_ < 0.05) due to DLK inhibition in nTg cerebellum, nTg forebrain, and Tg forebrain with relative gene expression changes (red = increased, blue = decreased) across genotype, treatment, and brain region for each sample (*n* = 6 samples/group). (**d**) Heatmap depicting the more robustly altered genes (*p*_adj_ < 0.05) due to DLK inhibition specifically in nTg cerebellum with relative gene expression changes (red = increased, blue = decreased) across genotype, treatment, and brain region for each sample (*n* = 6 samples/group). (**e**) Heatmap depicting the more robustly altered genes (*p*_adj_ < 0.05) due to DLK inhibition specifically in Tg forebrain with relative gene expression changes (blue = down; red = up) across genotype, treatment, and brain region for each sample (*n* = 6 samples/group). (**f**) Promoter analysis using DiRE of the most significantly (*p*_adj_ < 0.05) altered DLK-dependent genes (listed in (**c**–**e**)).

Since DLK signals through activation of transcription factors [50] we reasoned that changes in mRNA levels would identify differences in signaling output. We performed RNA-Seq on nTg forebrain, Tg forebrain, and nTg cerebellum of mice post continuous inhibition with 50 mg/kg DLKi or vehicle QD for seven days (Figure 7a). This dosing paradigm was chosen to allow for maximal effect on target mRNAs while minimizing potential large-scale changes in cell composition that could obscure the analysis. ANOVA and post-hoc tests accounting for brain region, genotype, and treatment identified significant (*p*_adj_ < 0.05) differentially expressed genes in the DLKi treatment groups (Figure 7b). In the nTg forebrain, only five genes were significantly affected by DLKi (Figure 7c); these were also altered similarly in Tg forebrain and nTg cerebellum and thus comprise a core set of DLK-dependent transcripts across brain regions and contexts. In contrast to this minimal response, the Tg forebrain exhibited 34 significant gene expression changes (Figure 7e), whereas 44 genes were differentially expressed in the nTg cerebellum (Figure 7d) for a total of 78 genes. The combined list of 78 genes were subjected to unsupervised clustering. Strikingly, other than the five core DLK responsive genes, all other responsive transcripts showed region-specific regulation and were significant only in the nTg cerebellum or Tg forebrain, but not both (Figure 7c–e). This entire list of 78 genes was submitted to regulatory element analysis to identify putative transcription factors mediating the response to DLKi, and Jun binding sites were the most significantly enriched, suggesting that p-c-Jun is the primary means by which DLK regulates transcription (Figure 7f). 

### 3.6. DLK Upregulates IGFBP5 Levels Specifically in the Cerebellum

**Figure 8:**
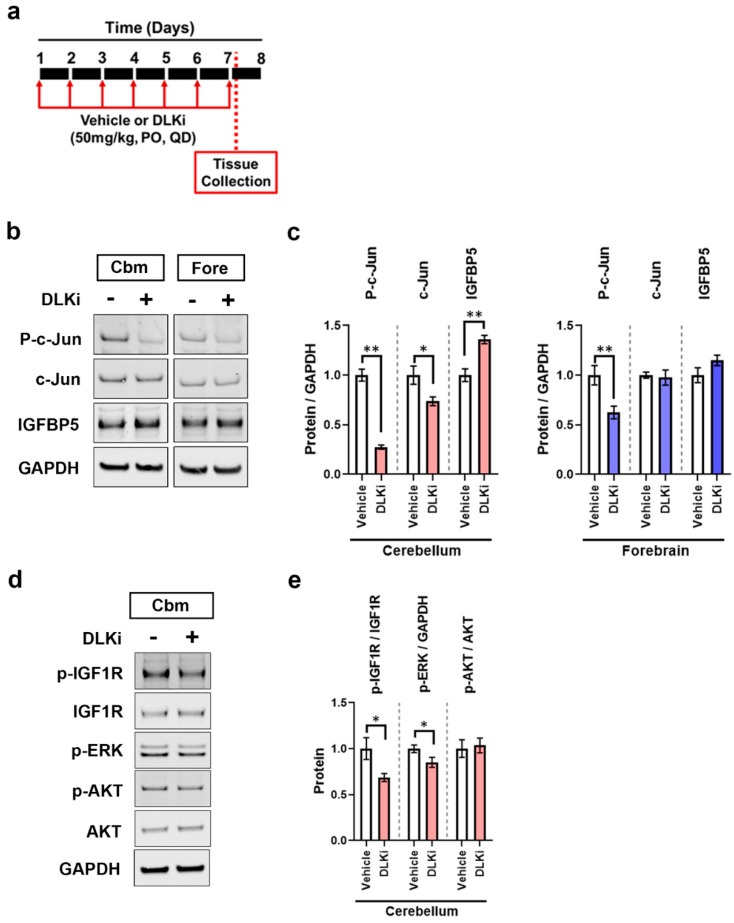
Sub-acute pharmacological inhibition of DLK modulates IGF1 binding protein-5 (IGFBP5) levels and IGF1 signaling in WT mice. (**a**) Schematic representation of study timeline; 3–4 months old male WT mice (*n* = 8) were treated with 50 mg/kg of DLKi or vehicle QD for 7 days. The study was terminated approximately 2 h post final dose. (**b**) Representative immunoblots of p-c-Jun, c-Jun, and IGFBP5 in cerebellum (Cbm) and forebrain (Fore) homogenates of WT mice treated with vehicle or DLKi. (**c**) Quantification of the immunoblots of p-c-Jun/GAPDH, c-Jun/GAPDH, and IGFBP5/GAPDH from cerebellum and forebrain homogenates of WT mice treated with vehicle or DLKi, plotted as mean fold change relative to vehicle group in respective brain region. Unpaired *t*-test. * *p* < 0.05, ** *p* < 0.01, *n* = 8. (**d**) Representative immunoblots of p-IGF1R, IGF1R, p-ERK, p-AKT, and AKT from cerebellum (Cbm) homogenates of WT mice treated with vehicle or DLKi. (**e**) Quantification of the immunoblots of p-IGF1R/IGF1R, p-ERK/GAPDH, and p-AKT/AKT from cerebellum homogenates of WT mice treated with vehicle or DLKi, plotted as mean fold change relative to vehicle group in respective brain region. * *p* < 0.05, unpaired *t*-test.

The function of constitutive DLK activity in adult brain homeostasis is unknown. Thus, we mined the RNA-Seq data to determine if any regulatory pathways were modulated in the cerebellum after DLK inhibition. One of the genes upregulated by DLKi in nTg cerebellum was insulin like growth factor binding protein 5 (*Igfbp5*), a key component of IGF1 signaling pathway, whereas two other components of the IGF1 signaling pathway, *Pik3r1* and *Pik3r3*, which encode for P85 and P55 subunits of phosphoinositide-3-kinase, were downregulated. To verify these findings, we dosed 3–4 months old male WT mice for seven days with 50 mg/kg of DLKi or vehicle QD, sacrificed the mice 2 h post final dose and performed Western blot analysis on the brain tissue (Figure 8a). p-c-Jun was decreased (*p* < 0.01) as expected in the cerebellum and as before, the low levels of p-c-Jun in the forebrain were also decreased (*p* < 0.01) (Figure 8b,c). As predicted from the RNA-Seq data, a significant induction of IGFBP5 (~40% increase, *p* < 0.01) was observed in DLKi-treated cerebellar homogenates compared to controls, but not in the forebrain. Thus, DLKi upregulates IGFBP5 protein specifically in the cerebellum, confirming the RNA-Seq observation. 

The predominant function of IGFBP5 is to limit the bioavailability of IGF1 and modulate its interaction with IGF1R. Binding of IGF1 to its receptor leads to phosphorylation of IGF1R, which further leads to phosphorylation and recruitment of the insulin receptor substrate-1 (IRS-1) and PI3K, consequently resulting in activation of downstream signaling components like AKT and MAPK pathways. Therefore, we analyzed cerebellar lysates from WT mice treated with 50 mg/kg of DLKi or vehicle QD for seven days for IGF1 signaling axis components. Phosphorylated-IGF1R (p-IGF1R) levels in the cerebellum were decreased by DLKi (*p* < 0.05) with no effect on total IGF1R (Figure 8d,e). Furthermore, DLKi decreased p-ERK (*p* < 0.05) but had no effect on p-AKT at the time points measured. 

**Figure 9:**
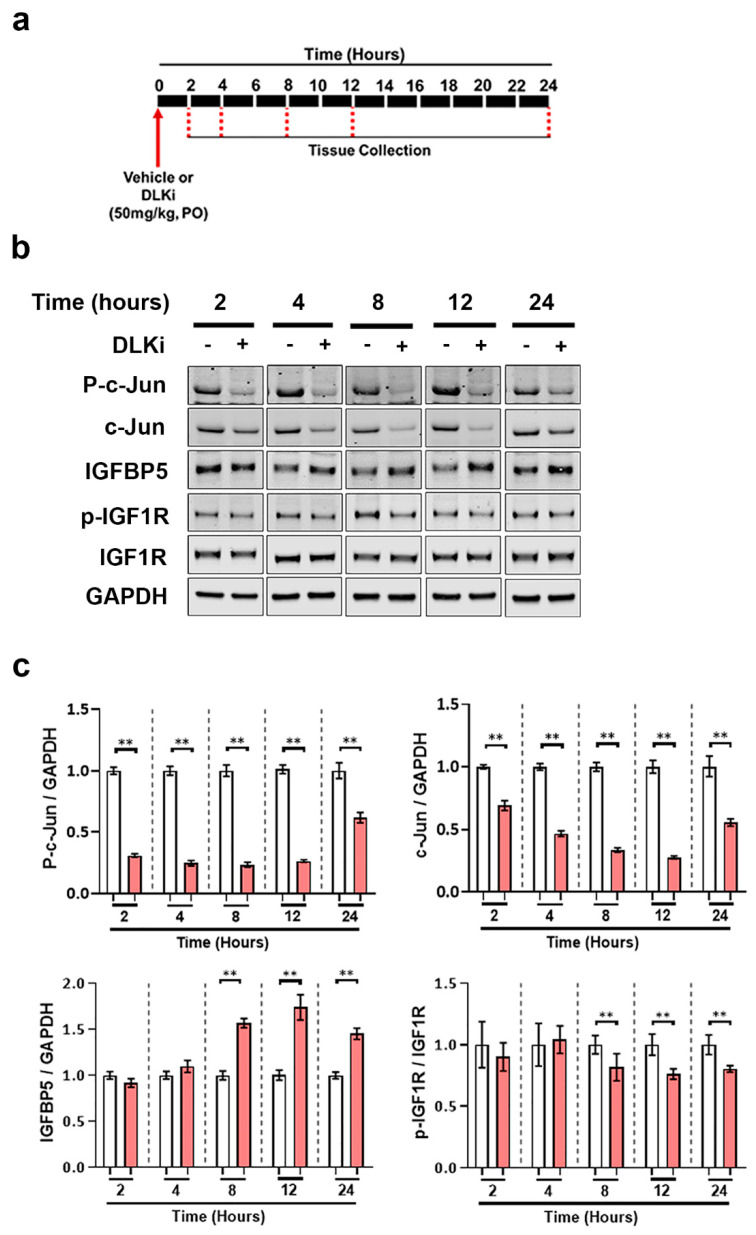
Acute pharmacological inhibition of DLK is sufficient to upregulate IGFBP5 in uninjured mouse cerebellum. (**a**) Schematic representation of study timeline; 3–4 months old male WT mice were treated with a single oral dose of 50 mg/kg of DLKi or vehicle QD. The study was terminated approximately 2, 4, 8, 12, or 24 h after single dose. (**b**) Representative immunoblots of p-c-Jun, c-Jun, IGFBP5, p-IGF1R, and IGF1R from cerebellar homogenates of WT mice treated with a single dose of vehicle or DLKi for either 2, 4, 8, 12, or 24 h. (**c**) Quantification of the immunoblots of p-c-Jun/GAPDH, c-Jun/GAPDH, IGFBP5/GAPDH, and p-IGF1R/IGF1R from cerebellar homogenates of WT mice treated with single dose of vehicle or DLKi for either 2, 4, 8, 12, or 24 h, plotted as mean fold change relative to vehicle group at respective time-point. Unpaired *t*-test. * *p* < 0.05, ** *p* < 0.01, *n* = 5.

To further understand the kinetics between DLK inhibition and upregulation of cerebellar IGFBP5, we performed Western blot analysis on cerebellar homogenates from adult male WT mice dosed with a single dose of 50 mg/kg of DLKi or vehicle and sacrificed at either 2, 4, 8, 12 or 24 h post dose (Figure 9a). DLKi decreased p-c-Jun levels by 2 h post administration, which was sustained for 24 h (Figure 9b,c). Western blot analysis showed that DLKi upregulated IGFBP5 (*p* < 0.01) 8 h post treatment, which was sustained through 24 h. This DLKi-induced IGFBP5 upregulation in the cerebellum was accompanied by a significant reduction of p-IGF1R. These data suggest that one function of constitutively active DLK in the adult brain is to promote IGF1 signaling in the cerebellum.

## 4. Discussion

The MAPK/JNK signaling pathway performs diverse, sometimes opposing functions, ranging from apoptosis to proliferation, depending on the context. Here we illustrate that this diversity extends to DLK, which is not only a regulator of neuronal development and injury response, but also performs homeostatic functions in the adult mouse cerebellum. We observed robust nuclear p-c-Jun in cerebellar granule cells in adult mice, and by blocking DLK with a highly selective inhibitor, p-c-Jun levels decreased rapidly. Given the highly selective nature of this DLKi (Figure S4a), cerebellar p-c-Jun is driven predominantly by DLK. 

This observation means that there must also be as-yet undefined processes that allow DLK to trigger p-c-Jun nuclear accumulation in cerebellar granule cells while restricting DLK-driven c-Jun phosphorylation in other types of neurons. This mechanism does not relate to DLK stabilization, as has been observed in mostly nerve injury models [12,19,24,25,26,51], because DLK levels were not induced in any condition examined. In fact, DLK’s direct substrate pMKK4 is suppressed by DLKi in both the forebrain and cerebellum regardless of disease status (Figure 4d,e), demonstrating that DLK is constitutively active in both brain regions, and that regulation occurs further downstream. We suspect this regulation occurs at the assembly of the signaling complex, because DLK/JNK scaffolding proteins (1) interact with motor proteins to allow transport of the signaling complex to the perinuclear area and (2) can recruit negative regulatory factors such as phosphatases that limit signaling [33]. 

Support for this idea comes from the regional distribution of JIP-1, JIP-3, and POSH. JIP-1 is more abundant in the cerebellum, whereas JIP-3 and POSH are more abundant in the forebrain, presumably leading to differences in DLK/JNK scaffolding. In dorsal root ganglion neurons, JIP-3, but not JIP-1, is essential for DLK-mediated apoptosis [52], highlighting the functional differences associated with the availability of specific scaffolding proteins. The strikingly higher levels of POSH in the forebrain relative to the cerebellum could also be functionally important. POSH can form a complex with JIP proteins [53] and itself is required for injury-induced hippocampal apoptosis [54,55]. Perhaps JIP-3 and POSH form heterodimers that limit DLK signaling via p-c-Jun in healthy hippocampal neurons but permit it during disease and injury. Future studies will be required to test this hypothesis. 

The comparative transcriptomic data revealed that DLK signaling can have significantly different outcomes depending on context. Other than a core set of five genes repressed in all conditions by DLKi, four of which are linked to the JNK pathway, there were no significant genes responsive to DLK inhibition in the non-transgenic forebrain (Figure 6). These data are consistent with the lower levels of p-c-Jun under normal conditions in that brain region. In contrast, DLK inhibition altered the expression of two entirely distinct gene sets in the nTg cerebellum and in the Tg forebrain. These transcriptional responses confirm that DLK is actively signaling in these contexts; in the cerebellum the signaling is constitutive and in the forebrain, it coincides with neuronal stress and injury. To illustrate this divergent signaling dependent on context, we chose the insulin growth factor-1 (IGF1) signaling pathway for further investigation. Based on the inhibition data, DLK normally represses IGFBP5 protein in the adult mouse cerebellum, promoting the phosphorylation of the IGF1 receptor and its signaling. The physiological role of IGF1 pathway modulation requires further exploration, but could relate to synaptic modulation, as DLK regulates synaptic strength in multiple contexts [15,16] as does IGF1 [56]. 

In summary, DLK is not only an injury response molecule but also performs homeostatic functions. Future studies aimed to understand how the signalsomes form and are trafficked in a context-dependent manner will provide greater insight into how this functional diversity is generated.

## Supplementary Materials

The following are available online at https://www.mdpi.com/1422-0067/21/14/4849/s1, Figure S1. Chemical structure of DLKi, Figure S2. Validation of DLK antibodies. (**a**) Double staining of DLK (green) and Hoechst (blue) on sagittal brain sections of WT DLK (+/+) and DLK (−/−) mice. (**b**) Immunoblot analysis of DLK using DLK antibodies from two vendors in whole brain lysates from DLK WT, DLK (+/−) and DLK (−/−) mice. Figure S3. DLK expression in adult uninjured mouse brain. (**a**) Representative immunoblot of DLK in lysates obtained from cerebellum (Cbm), anterior cortex (Ant Ctx), posterior cortex (Post Ctx), hippocampus (Hipp), striatum (Stm), and brainstem (Br St) of 3–4 months old male and female WT mice. (**b**) Quantification of immunoblots of DLK/GAPDH immunoblots in (**a**) (represented as fold change relative to cerebellar levels), Figure S4. Pharmacological inhibition of DLK inhibits constitutive as well as induced p-c-Jun in adult mouse brain. (**a**) The pharmacokinetic/pharmacodynamic relationship between total plasma DLKi exposure and % p-c-Jun/GAPDH reduction and total brain DLKi exposure and % p-c-Jun/GAPDH reduction measured in Figure 3a–c. The in vivo IC_50_ obtained from total plasma DLKi exposure and total brain DLKi exposure was 1.57 µM and 2.59 µM, respectively. (**b**) The pharmacokinetic/pharmacodynamic relationship between free plasma DLKi exposure and % p-c-Jun/GAPDH reduction and free brain DLKi exposure and % p-c-Jun/GAPDH reduction measured in Figure 3a–c. The in vivo IC_50_ obtained from free log plasma DLKi exposure and free log brain DLKi exposure was 0.207 µM and 0.176 µM, respectively. Concentration of DLKi plotted as logarithmic scale on the X-axis, Figure S5. Selectivity of DLKi in mouse brain. (**a**) Graphical representation of the kinases examined for kinome selectivity in 3–4 months old male WT mice dosed with single oral dose of DLKi by ActivX KiNativ analysis. (**b**) Percent DLK binding in 3–4 months old male WT mice brain tissue assessed by ActivX KiNativ assay plotted against the dose of DLKi (5 mg/kg (*n* = 2), 15 mg/kg (*n* = 2), and 50 mg/kg (*n* = 2)). (**c**) Percent DLK binding in 3–4 months old WT mice brain tissue assessed by ActivX KiNativ assay plotted against the total brain concentration of DLKi and free brain concentration of DLKi. Concentration of DLKi plotted as logarithmic scale on X-axis, Figure S6. Pharmacokinetic analysis of DLKi in brain at different time-points. (**a**) Schematic representation of study timeline; 3–4 months old male WT mice (*n* = 5) were dosed with single oral dose of 50 mg/kg of DLKi or vehicle. The study was terminated approximately 2, 4, 8, 12, or 24 h after single dose. (**b**) Plot of total brain concentration of DLKi and free brain concentration of DLKi against time (h) from mice dosed with single dose of DLKi. Dotted line represents IC50 of the DLKi, Figure S7. Differentially expressed genes in the MAPK pathway (>2-fold change) in nTg cerebellum versus nTg forebrain (padj < 0.05).
