# Supplementary material for: Dual Leucine Zipper Kinase Is Constitutively Active in the Adult Mouse Brain and Has Both Stress-Induced and Homeostatic Functions
Source: Int J Mol Sci. 2020 Jul 9;21(14):4849. doi: 10.3390/ijms21144849 (PMC7402291; doi:10.3390/ijms21144849)
Supplement: Supplementary file 1 [file ijms-21-04849-s001.pdf]

Supplementary Figures

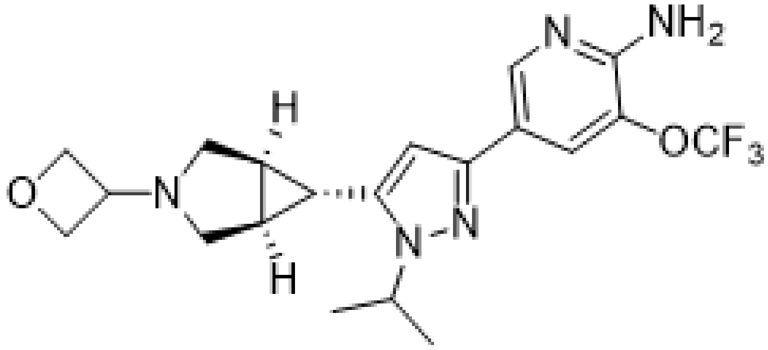

Figure S1. Chemical structure of DLKi.

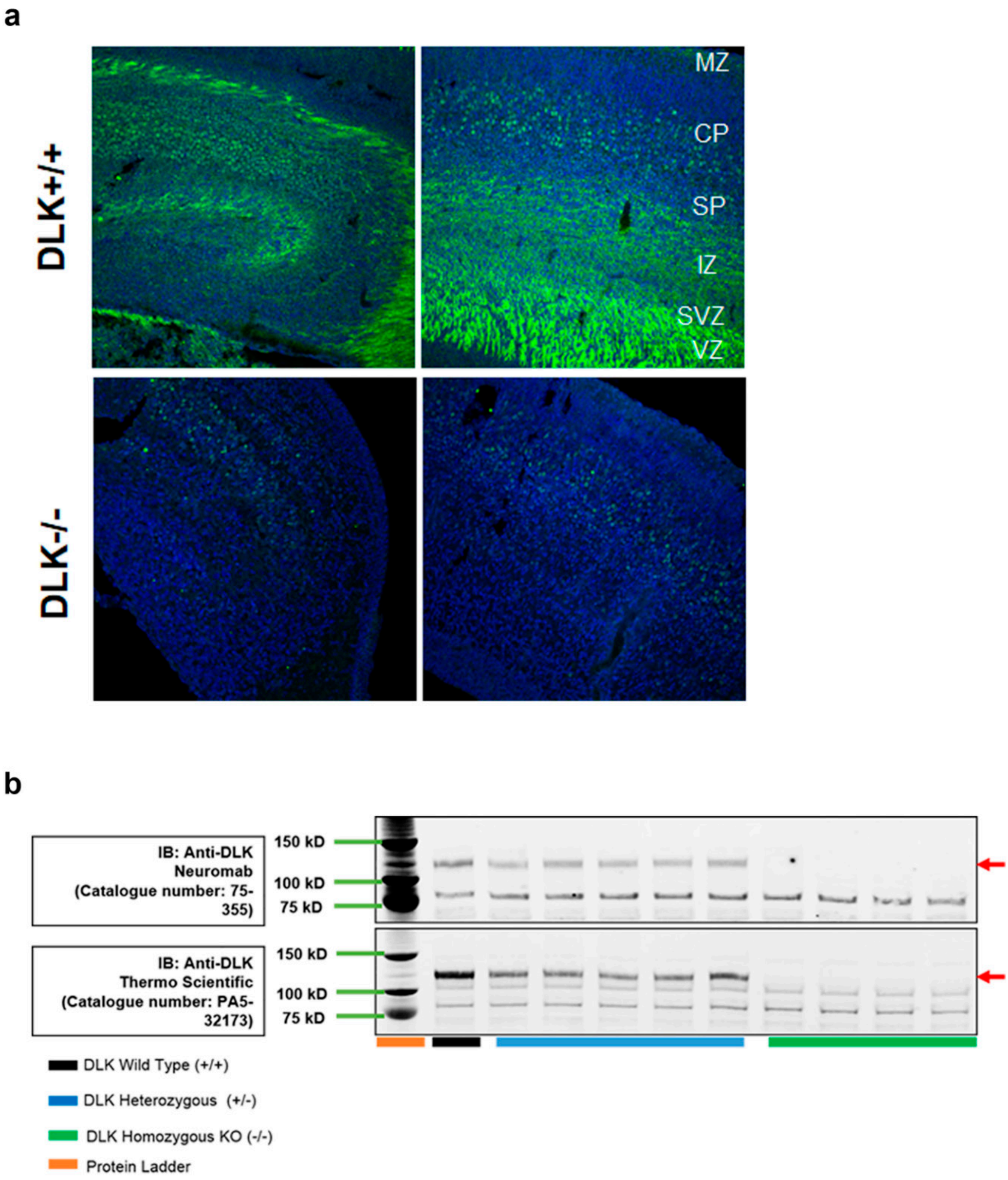

Figure S2. Validation of DLK antibodies. **(a)** Double staining of DLK (green) and Hoechst (blue) on sagittal brain sections of WT DLK (+/+) and DLK (-/-) mice. **(b)** Immunoblot analysis of DLK using DLK antibodies from two vendors in whole brain lysates from DLK WT, DLK (+/-), and DLK (-/-) mice.

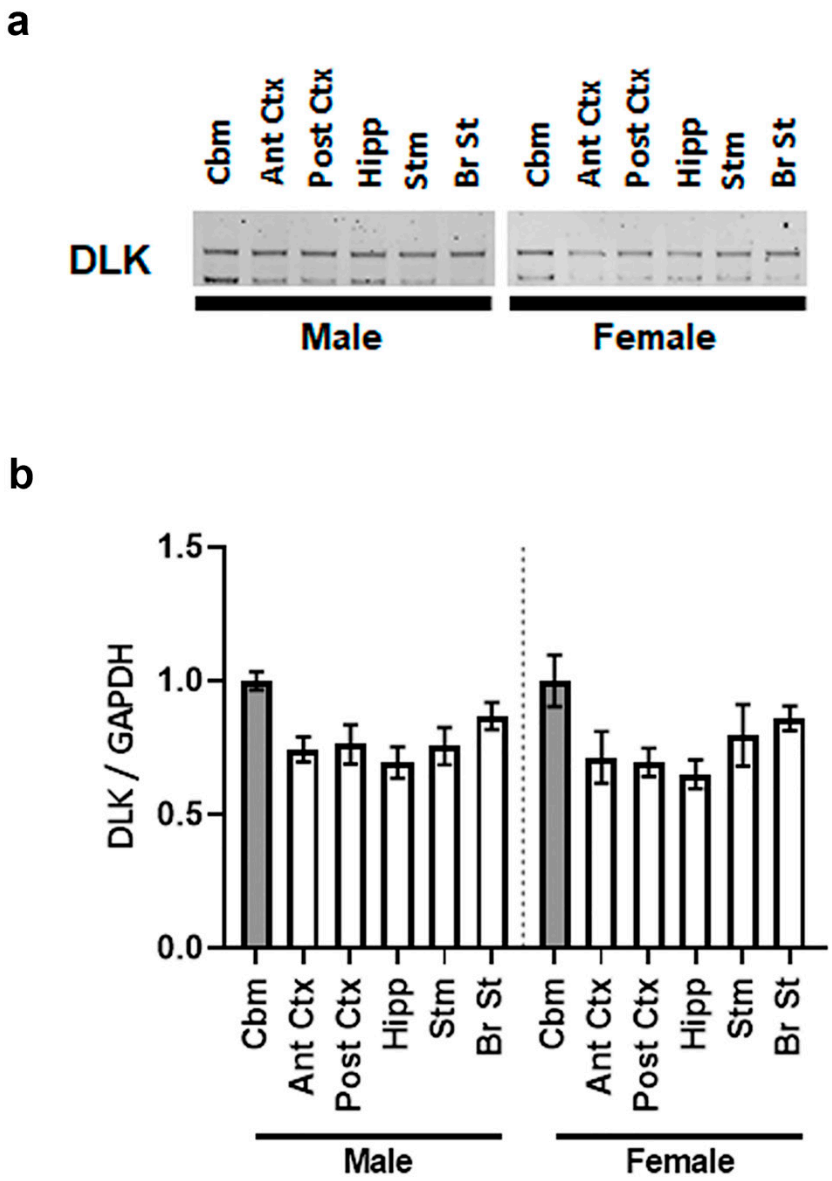

Figure S3. DLK expression in adult uninjured mouse brain. (**a**) Representative immunoblot of DLK in lysates obtained from cerebellum (Cbm), anterior cortex (Ant Ctx), posterior cortex (Post Ctx), hippocampus (Hipp), striatum (Stm), and brainstem (Br St) of 3–4 months old male and female WT mice. (**b**) Quantification of immunoblots of DLK/GAPDH immunoblots in (a) (represented as fold change relative to cerebellar levels).

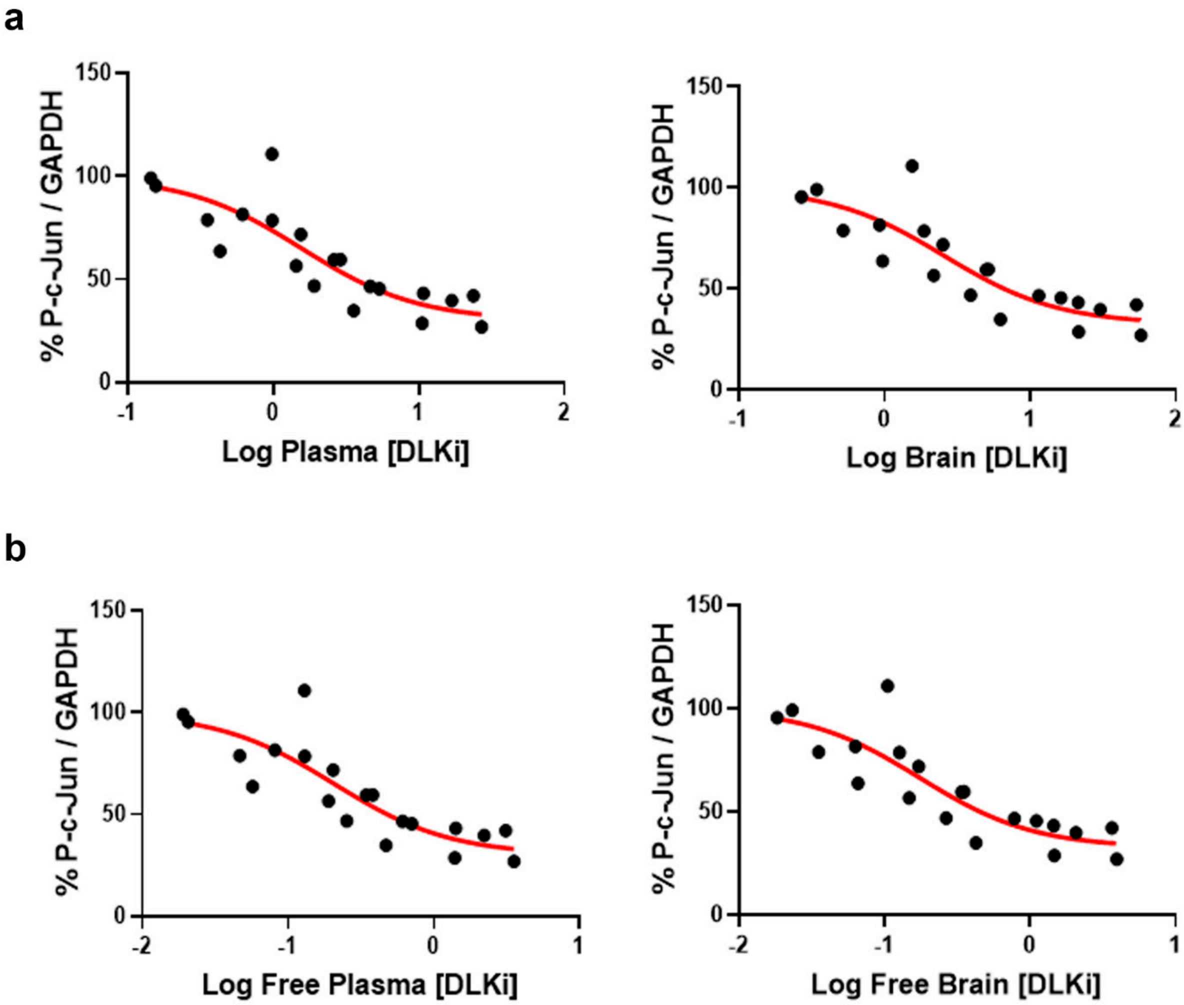

Figure S4. Pharmacological inhibition of DLK inhibits constitutive as well as induced p-c-Jun in adult mouse brain. (**a**) The pharmacokinetic/pharmacodynamic relationship between total plasma DLKi exposure and % p-c-Jun/GAPDH reduction and total brain DLKi exposure and % p-c-Jun/GAPDH reduction measured in Figure 3a–c. The in vivo IC_{50} obtained from total plasma DLKi exposure and total brain DLKi exposure was 1.57 μM and 2.59 μM, respectively. (**b**) The pharmacokinetic/pharmacodynamic relationship between free plasma DLKi exposure and % p-c-Jun/GAPDH reduction and free brain DLKi exposure and % p-c-Jun/GAPDH reduction measured in Figure 3a–c. The in vivo IC_{50} obtained from free log plasma DLKi exposure and free log brain DLKi exposure was 0.207 μM and 0.176 μM, respectively. Concentration of DLKi plotted as logarithmic scale on X-axis.

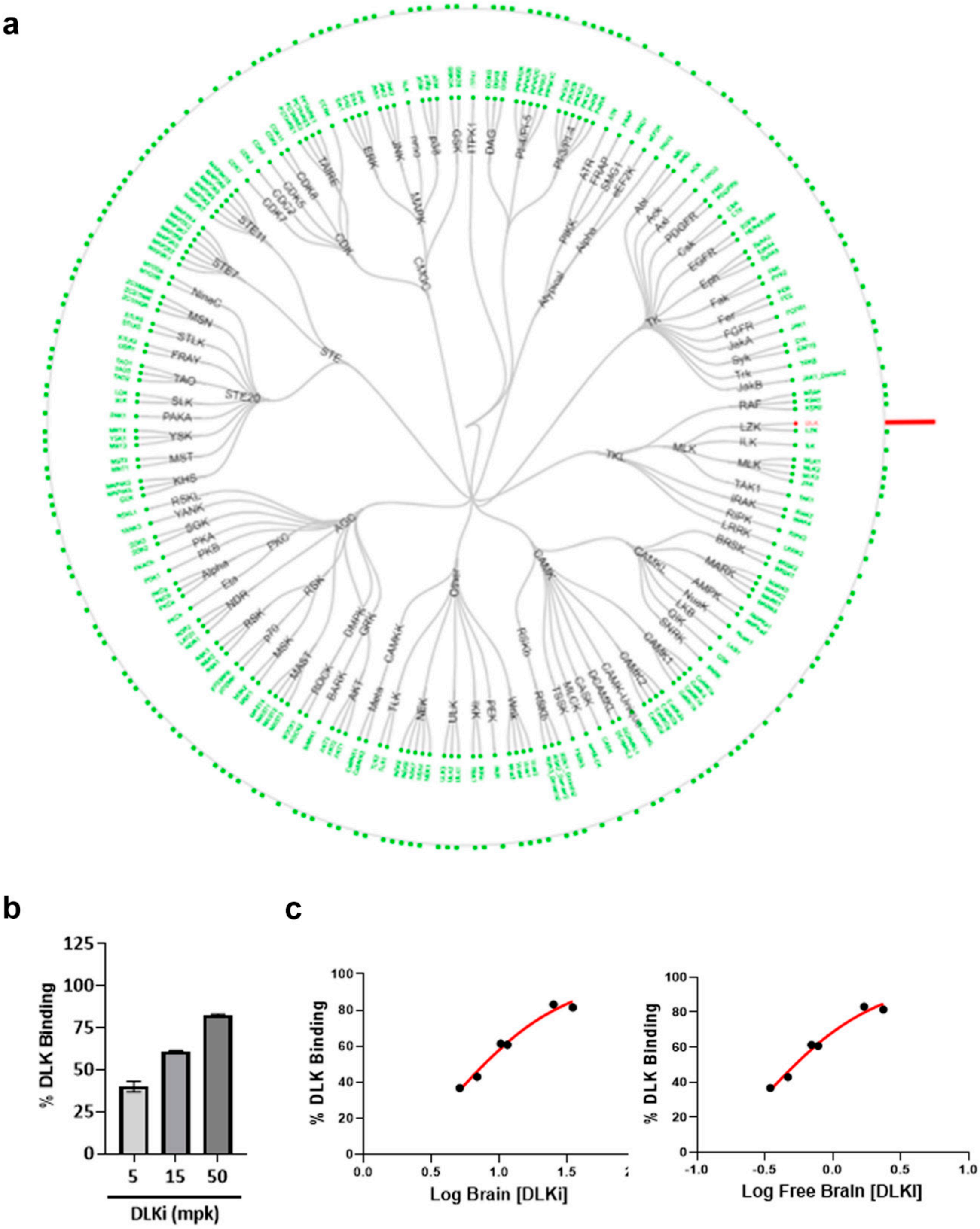

Figure S5. Selectivity of DLKi in mouse brain. (**a**) Graphical representation of the kinases examined for kinome selectivity in 3–4 months old male WT mice dosed with single oral dose of DLKi by ActivX KiNativ analysis. (**b**) Percent DLK binding in 3–4 months old male WT mice brain tissue assessed by ActivX KiNativ assay plotted against the dose of DLKi (5 mg/kg (*n* = 2), 15 mg/kg (*n* = 2), and 50 mg/kg (*n* = 2)). (**c**) Percent DLK binding in 3–4 months old WT mice brain tissue assessed by ActivX KiNativ assay plotted against the total brain concentration of DLKi and free brain concentration of DLKi. Concentration of DLKi plotted as logarithmic scale on X-axis.

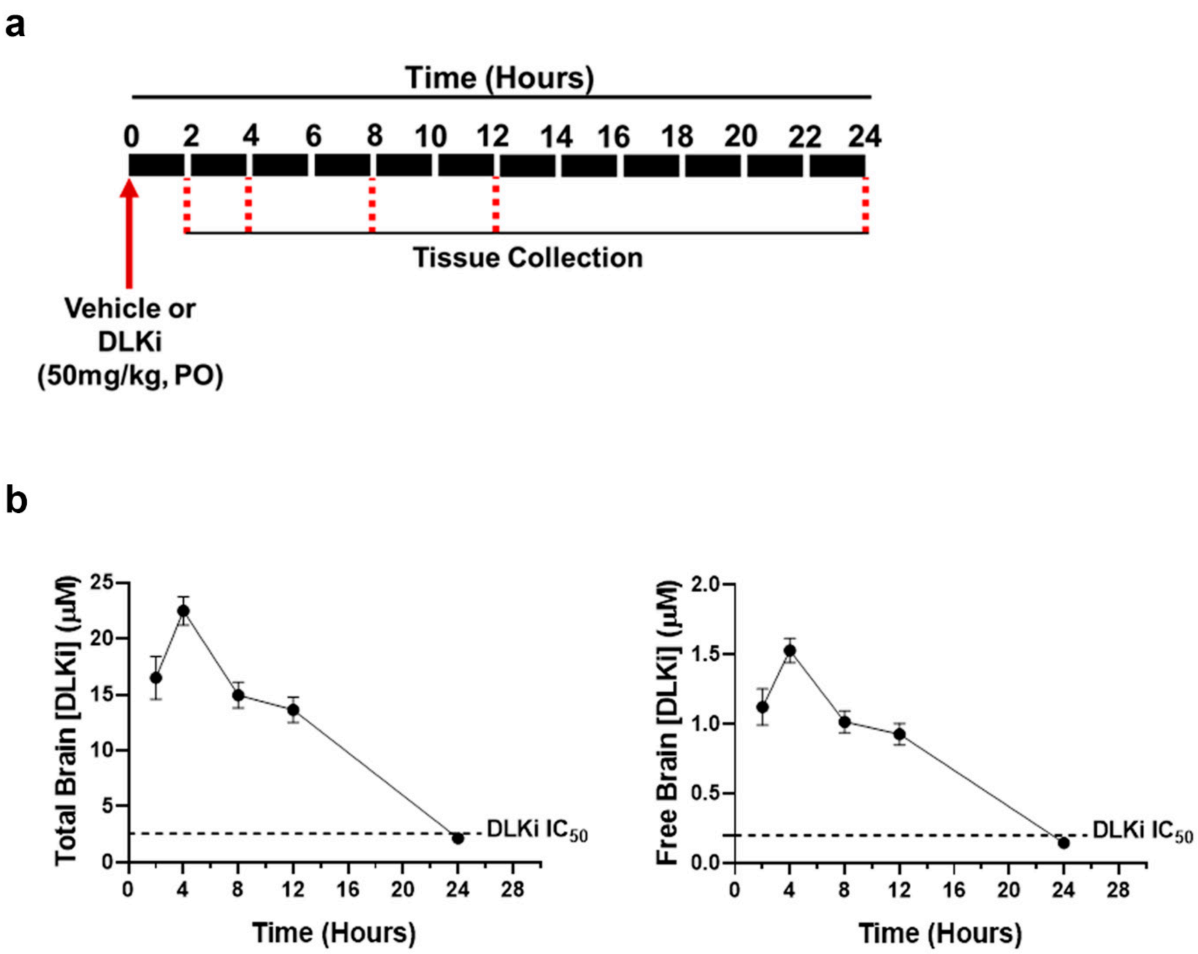

Figure S6. Pharmaokinetic analysis of DLKi in brain at different time-points. **(a)** Schematic representation of study timeline; 3–4 months old male WT mice (n = 5) were dosed with single oral dose of 50 mg/kg of DLKi or vehicle. The study was terminated approximately 2, 4, 8, 12, or 24 h after single dose. **(b)** Plot of total brain concentration of DLKi and free brain concentration of DLKi against time (h) from mice dosed with single dose of DLKi. Dotted line represents IC_{50} of the DLKi.

Symbol	Log2 FC	padj
Sh3rf1	-3.2	1×10^{-239}
Junb	-2.6	1×10^{-70}
Map3k15	-2.2	1×10^{-6}
Mapk15	-1.4	1×10^{-4}
Map3k19	-1.3	1×10^{-13}
Mapk1	-1.2	1×10^{-90}
Map3k21	-1.2	1×10^{-15}
Map4k1	-1.2	1×10^{-4}
Map9	-1.1	1×10^{-94}
Map2k1	-1.1	1×10^{-63}
Map4k2	1.0	1×10^{-66}
Map3k14	1.3	1×10^{-65}
Map3k8	1.3	1×10^{-23}
Map3k1	1.4	1×10^{-107}
Mapk6	1.5	1×10^{-168}
Mapk13	1.8	1×10^{-10}
Mapk12	3.6	1×10^{-287}

Figure S7. Differentially expressed genes in the MAPK pathway (>2-fold change) in nTg cerebellum versus nTg forebrain (padj < 0.05).
